# Significance of FXa and its receptor PAR2 for the growth of colon cancer cells *in vitro* and *in vivo*

**DOI:** 10.3389/fonc.2025.1631350

**Published:** 2025-07-21

**Authors:** Ulrike Meyer, Vincent Rönnpagel, Sophie Grammbauer, Mirjam von Lucadou, Ursula Rauch-Kröhnert, Edzard Schwedhelm, Frank Dombrowski, Christoph Ritter, Bernhard H. Rauch

**Affiliations:** ^1^ Pharmacology and Toxicology, University Medicine Oldenburg, Carl von Ossietzky Universität Oldenburg, Oldenburg, Germany; ^2^ Department of General Pharmacology, Institute of Pharmacology, University Medicine Greifswald, Greifswald, Germany; ^3^ Institute of Pathology, University Medicine Greifswald, Greifswald, Germany; ^4^ Institute of Clinical Pharmacology and Toxicology, University Medical Center Eppendorf, Hamburg, Germany; ^5^ Department of Cardiology, Angiology and Intensive Care, German Heart Center of Charité, Berlin, Germany; ^6^ Department of Clinical Pharmacy, Institute of Pharmacy, University Greifswald, Greifswald, Germany

**Keywords:** mouse model, colon cancer, PAR2 in colon cancer, FXa in colon cancer, *in vivo* study

## Abstract

Colon cancer is among the most common cancer types worldwide. Signaling pathways that control cell proliferation and migration play a crucial role in its progression. The G-protein-coupled protease-activated receptors (PARs) are associated mediators in this process. Both activated coagulation factors thrombin and FXa are capable of activating PARs. While thrombin, beyond its intrinsic role in hemostasis, primarily activates PAR1, FXa mediates its cellular effects independently via PAR2. Although the role of thrombin and PAR1 activation in cancer development has been established for some time, the impact of FXa-PAR2 on tumor progression represents a relatively novel area of investigation. Therefore, the current study was conducted to examine the role of FXa and PAR2 signaling in colon cancer progression using the murine colon cancer cell line MC38. Proliferation and migration assays were performed in vitro and signaling pathways analyzed by Western blot. In vivo, tumor growth and health status were investigated in WT and PAR2-KO mice. The findings demonstrate that FXa considerably augments the proliferation and migration of colon cancer (CC) cells *in vitro*. A molecular mechanism of action has been identified in the activation of PAR2 by FXa. The coagulation factor significantly induces MAPK- and AKT-signaling with EGFR transactivation in the murine MC38 cells utilized. Although oral treatment with a direct FXa inhibitor (Apixaban) at a dosage of up to 50 mg/kg did not significantly affect tumor growth *in vivo*, PAR2 deficiency resulted in significantly reduced tumor growth and enhanced health condition status, indicating a key role of PAR2 in the progression of colon cancer.

## 1 Introduction

According to the International Agency for Research on Cancer (IARC), the specialized cancer agency of the WHO, colorectal cancer is the second most prevalent cause of mortality among males and females on a global scale. In 2022, the global mortality rate from this neoplastic disease was almost 1 million (1). The incidence of malignant colon changes is increasing in emerging countries, where the risk of colon cancer was previously relatively low, on a global scale (2). The WHO attributes this increase to poor lifestyle choices by individuals (3). Diet and an unhealthy lifestyle are responsible for approximately one-third of all tumor cases and almost 55% of the colon cancer cases (3–5).

Malignant intestinal tissue differs from healthy tissue, in part due to the upregulation of protease-activated receptor-1 (PAR1). PAR1 is upregulated in inflamed colons (6). Its activation increases proliferation and migration of colon epithelial cells by activating extracellular signal-regulated protein kinases 1 and 2 (ERK1/2), also known as p44/42 MAPK. Moreover, PAR2 activation, along with PAR1, may play a vital role in ERK1/2 phosphorylation in colon cancer cells, which possibly leads to pro-migratory and pro-proliferative effects (7, 8). The transactivation of the epidermal growth factor receptor (EGFR) seems to have a significant effect in this process. The transmembrane tyrosine kinase induces the growth of colon cancer following PAR2 signaling (7). Compared to PAR1, PAR2 is highly expressed in the gastrointestinal tract (9).

The protease activated receptors, PAR2 and PAR1, share a unique mechanism of activation. The receptors belong to a subfamily of G protein-coupled receptors that contain a tethered ligand at the N-terminus. After terminal cleavage by serine proteases, the cryptic ligand is unmasked and binds to the second extracellular loop, causing the receptor to undergo self-activation. In addition to trypsin or matrix metalloproteinases, the proteolytically active coagulation factors thrombin and FXa can trigger PAR activation (10–13). Thrombin activates PAR1, PAR3 and PAR4 while FXa mediates its cellular effects especially via PAR2 (14). Additionally, synthetic peptides that have sequence homology to the bound ligand, known as PAR-activating peptides (AP), can activate the receptor without prior proteolytic cleavage (12, 15).

As cancer is often associated with an impaired coagulation status this significantly increases the risk of thromboembolic events over time. These events serve as significant predictors of reduced survival within the first year (16). Conversely, it has been observed that 20% of patients requiring antithrombotic treatment have cancer (17). In this context, the impact of PAR signaling on tumor progression has probably been rather underestimated to date. Given the high prevalence and mortality associated with colon cancer, there is an urgent need to develop novel approaches to diagnosis, treatment and prevention based on a more profound comprehension of the molecular mechanisms underlying this disease. Therefore, the current study was conducted to examine the correlation between tumor progression and PAR2 signaling in colon cancer both *in vitro* and *in vivo*.

## 2 Materials and methods

### 2.1 Cell culture

The murine colon cell line MC38 was purchased by Kerafast [CVCL_B288, Cat.#ENH204-FP]. These cells are derived from a colon adenocarcinoma of epithelial origin from a female mouse. The MC38 cell line was maintained in RPMI 1640 medium [PAN Biotech, Cat. #P04-17500] with 10% FCS [PAN Biotech, Cat. #PANBP30-3306] and 2 mmol/l L-glutamine [PAA Laboratories, Cat. #M11-006] and utilized until the 27th passage. The cell line was monthly tested for mycoplasma contamination via PCR analysis [Abcam, Cat. #ab289834].

Cells were stimulated with FXa (0,1 — 30 nM) [Enzo Lifescience, Cat. #BML-SE362-0100], thrombin (0,1 — 10 U/ml) [Enzo Lifescience, Cat. #BML-SE363-1000], selective PAR1-activating peptide AP1 (sequence: SFLLRN, 10 — 100 µM) [Bachem, Cat. #H-6416] or selective PAR2-activating peptide AP2 (sequence: SLIGRL, 10 — 100 µl) [Bachem AG, Cat. #H-5078] in a time- dependent manner after maintaining the cells in serum-free media for 16 h. For the investigation of an EGFR involvement, Erlotinib was used in ascending concentrations (50 nM - 1 µM).

### 2.2 Proliferation assay

Cell counts were quantified using a CASY TT Cell Counter & Analyzer [RRID: SCR_002080, Omni Life Science] and cell proliferation was quantified utilizing a BrdU (5-bromo-2’-deoxyuridine) cell proliferation kit [Merck, Cat. #2750]. Cells were stimulated with the respective stimuli and proliferation was assessed at 24, 48 and 72 hours. The BrdU assay was carried out according to the manufacturer’s instructions and measured using the multiplate reader Tecan Infinite M200 [RRID: SCR_024560, Tecan Group]. The magnitude of the absorbance is proportional to the quantity of incorporated BrdU, directly reflecting cell proliferation.

### 2.3 Migration assay

The experimental procedures were performed as previously described (18).

#### 2.3.1 Wound scratch assay

Undirected cellular migration was investigated by a wound healing assay. The wound was created by scratching the confluent cell monolayer with a 100 µl pipette tip. Following two washing steps, the cells were incubated in culture medium containing 5 mM hydroxyurea [Merck, Cat. #H8627] to fully suppress cell proliferation. The scratched wound was imaged using PALMRobo Software [RRID: SCR_014435] of AxioVision Imaging system [RRID: SCR_002677, Carl Zeiss Microscopy]. The precise location of the images was saved to analyze the same area post-incubation with the test stimuli. Following pre-incubation with hydroxyurea (1 mM) for one hour, the cells were treated with varying concentrations of FXa, thrombin, AP1 or AP2 in a hydroxyurea-containing medium for 24 hours.

#### 2.3.2 Boyden chamber assay

Directed migration was determined utilizing the Boyden chemotaxis chamber [Neuro Probe Inc.]. 5 × 10^3^ cells per well were placed in serum-free medium in the upper compartment, whilst the lower one was filled with the stimulus, separated by a polycarbonate membrane with a pore size of 8 μm [Whatman GmbH, Cat. #10400112]. The membrane was pre-coated with collagen for 24 hours at room temperature and then washed with PBS and dried. Following a 3-hour migration period, the cells were fixed on the membrane’s lower side with 4% PFA [Carl Roth, Cat. #0335.1] and subsequently stained with crystal violet solution [Merck, Cat. #V5265] and counted using ImageJ software [RRID: SCR_003070, National Institute of Health]. All treated samples were normalized to the untreated control cells (100%). Erlotinib (161 nM); SB203580 (0,5 µM) [Merck, Cat. #S8307], Ly29402 (20 µM) [Merck, Cat. #440202] and PD98059 (10 µM) [Merck, Cat. #P215] were employed to ascertain the impact on the migratory capacity of the cells.

### 2.4 Drug cytotoxicity assay

Drug cytotoxicity was assessed utilizing resazurin- and crystal violet-based assays [Serva Electrophoresis, Cat. #39905]. After a 16-hour starvation period, Erlotinib-containing medium was added to the cells. The cells were fixed with 4% PFA before being stained with a crystal violet-ethanol mixture (3.6:1). The cells were subsequently washed several times until the dye ceased to appear. Incubating 100 µl SDS (1%) per well on a rocking shaker dissolved the dye. The absorption was measured at 560 nm using Tecan Infinite M200 [RRID: SCR_024560, Tecan Group]. All treated samples were normalized to the untreated control cells (100%). EC_50_ values were estimated from dose-response curves using GraphPad Prism [RRID: SCR_002798, GraphPad].

### 2.5 RT qPCR

RNA was extracted using TRIzol Reagent [Thermo Fisher Scientific, Cat. #15596018]. High-Capacity cDNA Reverse Transcription Kit [Applied Biosystems, Cat. #4374967] was used to reversely transcribe the RNA. In 20 µl total volume, Blue Probe qPCR Kit [Biozym Scientific, Cat. #331456XL] was used to perform the subsequent qRT-PCR in duplicates on a Bio-Rad C1000 thermal cycler [RRID: SCR_019688, Bio-Rad Laboratories]. Relative quantities (Δ cycle threshold values) were obtained by normalization against 18S or GAPDH. The utilized Assays on demand [Applied Biosystems, Cat. #4331182] were used: Mm00433160_m1, Mm00438851_m1, Mm00439498_m1, Mm00445021_m1, Mm004488841_g1, Mm00468695_s1, Mm00473016_m1, Mm00476227_m1, Mm00486079_m1, Mm02619656_s1, Mm02620181_s1, Mm02620565_s1, Mm03039020_m1, Mm99999915_g1, Mm03928990_g1, Mm00731567_m1. Expression levels measured by qPCR were calculated using DDCt method. The Ct value of the target is determined by comparison to the Ct value of the reference gene (19).

### 2.6 Western blot analysis

Cells were dissolved in lysis buffer (50 mM Tris-HCl, 100 mM NaCl, 0.1% Triton X-100, 5 mM EDTA, pH 7.4) using a fresh protease inhibitor cocktail [Merck, Cat. #539131] on ice. Protein concentrations were determined by BCA assay [Thermo Fisher, Cat. #23225]. Following denaturation in Laemmli SDS sample buffer, 20 μg protein were separated on 10% SDS polyacrylamide gels. After electrophoretic separation using SDS-PAGE, the proteins were transferred to nitrocellulose membranes [Thermo Fisher, Cat. #88018]. After blocking [Li-cor, Cat. #927-60001], membranes were incubated overnight at 4°C under rotating conditions with the following primary antibodies: AKT (1:1000) [Cell Signaling, Cat. #4691], phospho-AKT (1:1000) [Cell Signaling, Cat. #4060], p38 (1:500) [Cell Signaling, Cat. #8690], phospho-p38 (1:500) [Cell Signaling, Cat. #4511], p44/42 (1:1000) [Cell Signaling, Cat. #4695], phospho-p44/42 (1:1000) [Cell Signaling, Cat. #4370], PAR2 (1:200) [Santa Cruz Biotechnologies, Cat. #sc-13504], PAR1 (1:200) [Santa Cruz Biotechnologies, Cat. #13503]. The membranes were washed three times with TBST for five minutes each, followed by incubation with one of the following fluorescence-labeled secondary antibodies: Donkey Anti Goat IRDye^®^ 800 (1:20.000) [RRID: AB_621846, Li-cor, Cat. #926-32214], Goat Anti Rabbit IRDye^®^ 800 (1:20.000) [RRID: AB_2651127, Li-cor, Cat. #926-32211], Goat Anti Mouse IRDye^®^ 800 (1:20.000) [RRID: AB_621842, Li-cor, Cat. #926-32210]. After rinsing the membranes with TBST at room temperature on a horizontal shaker three times for five minutes each, fluorescence signals were detected using Odyssey^®^ CLx Imaging System [RRID: SCR_014579, Li-cor]. As a loading control, total protein staining was performed following the manufacturer’s instructions [Li-cor, Cat. #926-11011].

### 2.7 *In vivo* tumor model

To investigate colon cancer cell growth *in vivo*, the MC38 cells (1x10^6^) were subcutaneously injected into both male and female C57BL/6 wild-type (WT) mice with a minimum of 18 g weight [RRID: IMSR_JAX:000664, Jackson laboratories]. To ascertain the direct impact of PAR2 on tumor progression, commercially available PAR2 knockout (PAR2-KO) mice with a C57BL/6J background and a minimum of 18 g weight were employed as a comparison group [RRID: IMSR_JAX:004993, Jackson laboratories]. The protocol for generating the PAR2-KOstrain is described by Schmidlin et al. (20) The PAR2 knockout was confirmed by genotyping (detailed information in the **Supplementary Data**) The mice, aged 8 to 10 weeks, were divided into three groups: control (vehicle only) and 2 Apixaban treatment groups (including vehicle). Each group comprised 9–11 mice. To prevent the formation of family-related clusters of effects, care was taken to ensure that siblings were distributed across different groups (detailed information can be found in the supplementary part of the manuscript). Daily oral administration of either 5 mg/kg body weight or 50 mg/kg body weight of the direct FXa inhibitor Apixaban [provided by Bristol-Myers Squibbs] or vehicle only [Gattefossé, Cat. #M 2125 CS] was given. Tumor growth was observed within 21 days after cell injection. Subsequently, a tail bleeding assay was conducted. The mouse tail was clipped and placed in a sodium chloride solution at 37°C, with the time taken to reach hemostasis measured.

Ethics statement: The corresponding animal study proposal was reviewed and approved by the local authority office for animal studies, the “Landesamt für Landwirtschaft, Lebensmittelsicherheit und Fischerei Mecklenburg-Vorpommern” No. 7221.3-1.1-013/18.

Resected tumors were fixed in 4% formalin [Sigma-Aldrich, Cat. #47608] for 24 h at room temperature. The tissues were then subjected to a dehydration process and embedded in paraffin. Tissue sections from the tumors were prepared at predetermined depths and mounted on slides for the subsequent staining procedures. Haematoxylin and eosin (H&E) staining was performed according to the manufacturer’s protocol by Sigma-Aldrich [Mayers Hämalaunlösung, Cat. #1.09249; Eosin-Methylenblaulösung, Cat. #1.01383]. Staining with Pikro-Sirius red staining was carried out using the Morphisto staining kit according to the manufacturer’s instructions [Morphisto, Cat. #13425.00100].

### 2.8 UPLC-MS/MS

Apixaban was quantified by ultra-performance liquid chromatography coupled with tandem mass spectrometry (UPLC-MS/MS) as described previously with minor modifications (21). In brief, 20 µl of each sample were added to 180 µl of the internal standard 13C^2^, H_3_-apixaban (0.1 µM resolved in methanol, Toronto Research Chemicals, Toronto, ON, Canada). After protein precipitation and centrifugation, 40 µl of the supernatant were collected, diluted with 100 µL of ultra-pure water and subjected to ultra-performance liquid chromatography on an AQUITY UPLC BEH C8 1.7 µm column (2.1x75 mm, Waters, Eschborn, Germany). Apixaban was eluted with a binary gradient of 0.1% formic acid in acetonitrile and 0.1% formic acid in water at a flow rate of 0.5 mL/min for 4.6 minutes and subsequently, Apixaban (retention time 2.7 min, m/z 460.21) was fragmented to its daughter ion m/z 443.19 while 13C^2^, H_3_-apixaban (m/z 464.28) was fragmented to m/z 447.19 on a Xevo Triple Quadrupole Mass Spectrometer (Waters). Calibration curves in mouse plasma were generated to calculate absolute Apixaban concentrations in samples. The limit of detection was 1 ng/mL Apixaban.

### 2.9 Statistical analysis

Statistical analyses were conducted using GraphPad Prism software 8.0 [RRID: SCR_002798, GraphPad, Boston, USA]. Mean ± SD of n independent experiments is presented for all data. D’Agostino-Pearson, Shapiro-Wilk and Kolmogorov-Smirnov tests were employed to ascertain the normal distribution of the data. Three or more groups were tested for statistical significance using analysis of variance (ANOVA). Dose-response curves and ED_50_ values were derived through non-linear regression modelling (log(inhibitor) vs. normalized response - Variable slope). The results were considered significant at p < 0.05 (*).

## 3 Results

### 3.1 FXa and selective activation of PAR2 significantly affect proliferation and migration of murine colon cancer cells *in vitro*

**Figure 1:**
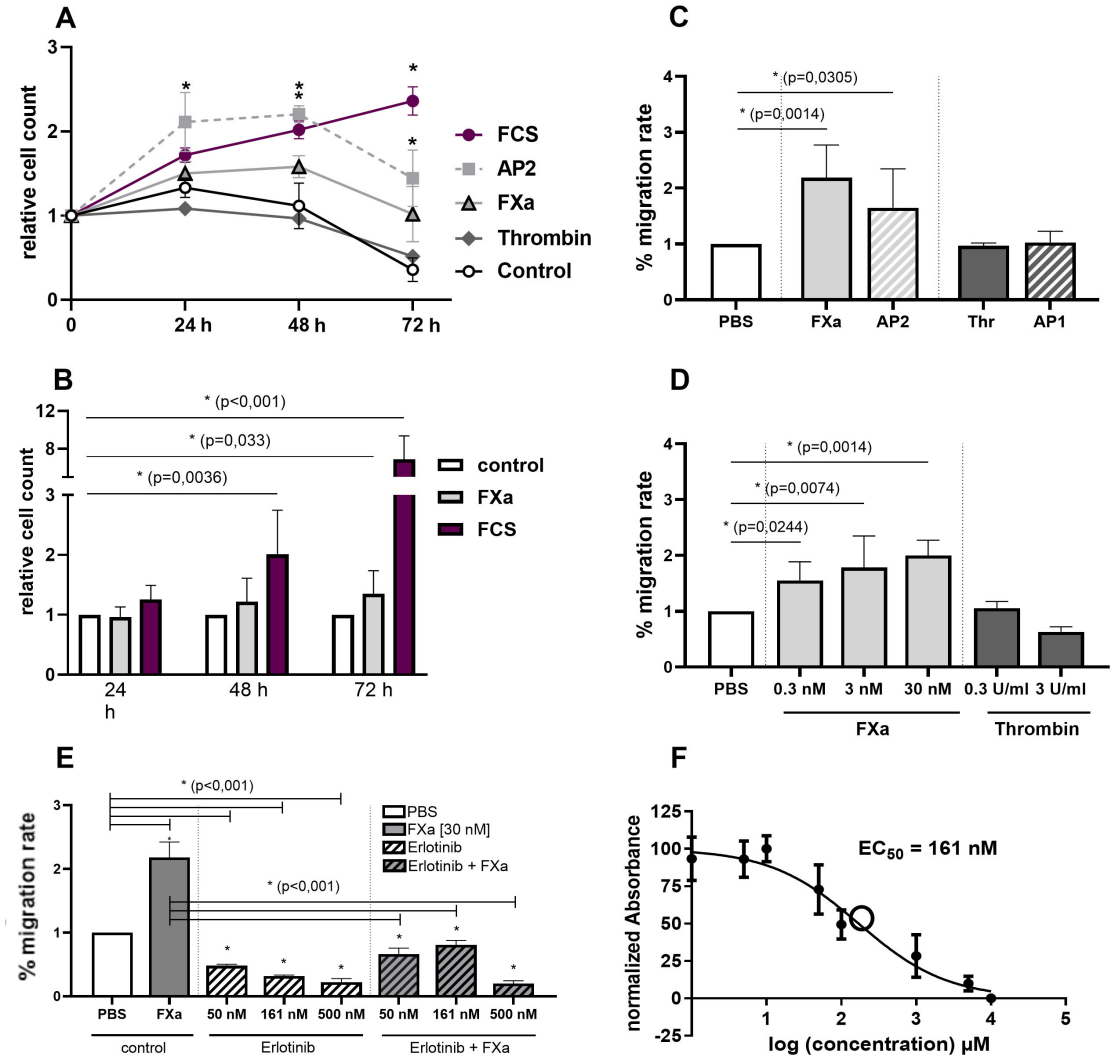
*In vitro* effects of FXa and PAR2 activation on proliferation and migration of murine CC cell line MC38 and involvement of EGFR. Proliferation. **(A)** Selective activation of PAR2 using AP2 induces significant proliferation, with the strongest stimulus being 10% FCS in the medium. Also, longterm stimulation with FXa enhances proliferation of the MC38 cell line. **(B)** Comparative analyses using BrdU assay exhibit equivalent increased proliferation rates after FXa stimulation of the cells. Migration. **(C)** Directed and **(D)** undirected cell migration of murine CC cells are significantly increased by FXa stimulation and selective PAR2 activation. Mean ± SD of n independent experiments is presented for all data. **(A)** n =7-11, **(B)** n =3-5, **(C)** n =5-7, **(D)** n =3, One-way ANOVA, Dunnett posthoc test, p < 0.05 (*). EGFR. **(E)** The involvement of EGFR was analyzed by pre-incubation of the cells with Erlotinib, an EGFR inhibitor, at varying concentrations and partially FXa stimulation. EGFR inhibition significantly decreased migration of the cell line used. Mean ± SD of n independent experiments is presented for all data. n =3-6, One-way ANOVA, Dunnett posthoc test, p < 0.05 (*). **(F)** The half-maximal inhibitory concentration (IC_50_) of the EGFR-inhibitor, Erlotinib, was determined as 161 nM utilizing a resazurin-/crystal violet assay, n =3-6, Dose-response curves and ED50 values were derived through non-linear regression modelling (log(inhibitor) vs. normalized response - Variable slope).

The measured basal doubling time of the murine MC38 cell line is around 20 hours. After incubation with coagulation factor FXa at concentrations of up to 30 nM in a time-dependent manner (24 h — 72 h), the growth rate of the MC38 cells was significantly elevated at 72 h when compared to the negative control without FXa (**Figure 1A**). Previous studies from our group indicated that FXa is generated at physiological concentrations of up to 30 nM within a blood clot and is subsequently released from the clot over time (22). Therefore, 30 nM FXa was adopted as the standard concentration for further experiments. The negative control was achieved through the use of starvation medium lacking FCS, whilst a positive control was established with the inclusion of 10% FCS. FCS proves to be a very potent growth stimulant due to its content of essential nutrients, adhesion factors and growth factors.

BrdU assay conducted for comparative analysis indicate substantially enhanced proliferation rates following FXa stimulation (**Figure 1B**). This effect was mimicked and potentiated through selective PAR2 activation via AP2 (**Figure 1A**). In contrast, thrombin displayed no significant response in the murine CC cell line. Moreover, FXa considerably affected the migration of the cancer cells. Physiological concentrations of FXa significantly enhance undirected (**Figure 1C**) and directed (**Figure 1D**) migration of MC38 cells. Both of these phenomena crucially participate in the pathogenesis of carcinomas. Cell stimulation with FXa significantly increases cell migration, whereas thrombin or selective peptide-induced activation of PAR1 did not induce a noteworthy response (**Figures 1C, D**). Stimulation with the PAR2-activating peptide AP2 mimics the effect of FXa (**Figure 1C**). Furthermore, in human CC cells, FXa exerts a substantial pro-migratory effect on the cells, a phenomenon that can be mimicked through selective PAR2 activation (see **Supplementary Figure S3**). Subsequently, the involvement of the epidermal growth factor receptor (EGFR) in controlling migration was examined by pre-incubating the cells with Erlotinib, an EGFR inhibitor, at various concentrations. The findings indicated that the inhibition of the EGFR significantly reduced the migration of the cancer cells, and that subsequent stimulation with FXa was unable to reverse this effect (**Figure 1E**). The half-maximal inhibitory concentration (IC_50_) of the inhibitor was calculated in advance using a resazurin/crystal violet assay, which resulted in a concentration of 161nM (**Figure 1F**).

### 3.2 Activation of mitogenic and migratory signaling pathways by FXa and EGFR transactivation

To demonstrate the potential mechanism underlying the influence of FXa on the proliferation and migration of murine MC38 cell line, Western Blot analyses were conducted to examine activated signaling pathways. The results indicate that FXa prompts an activation of mitogenic and migratory pathways, suggesting a direct response of colon cancer cells to coagulation factor signaling.

**Figure 2:**
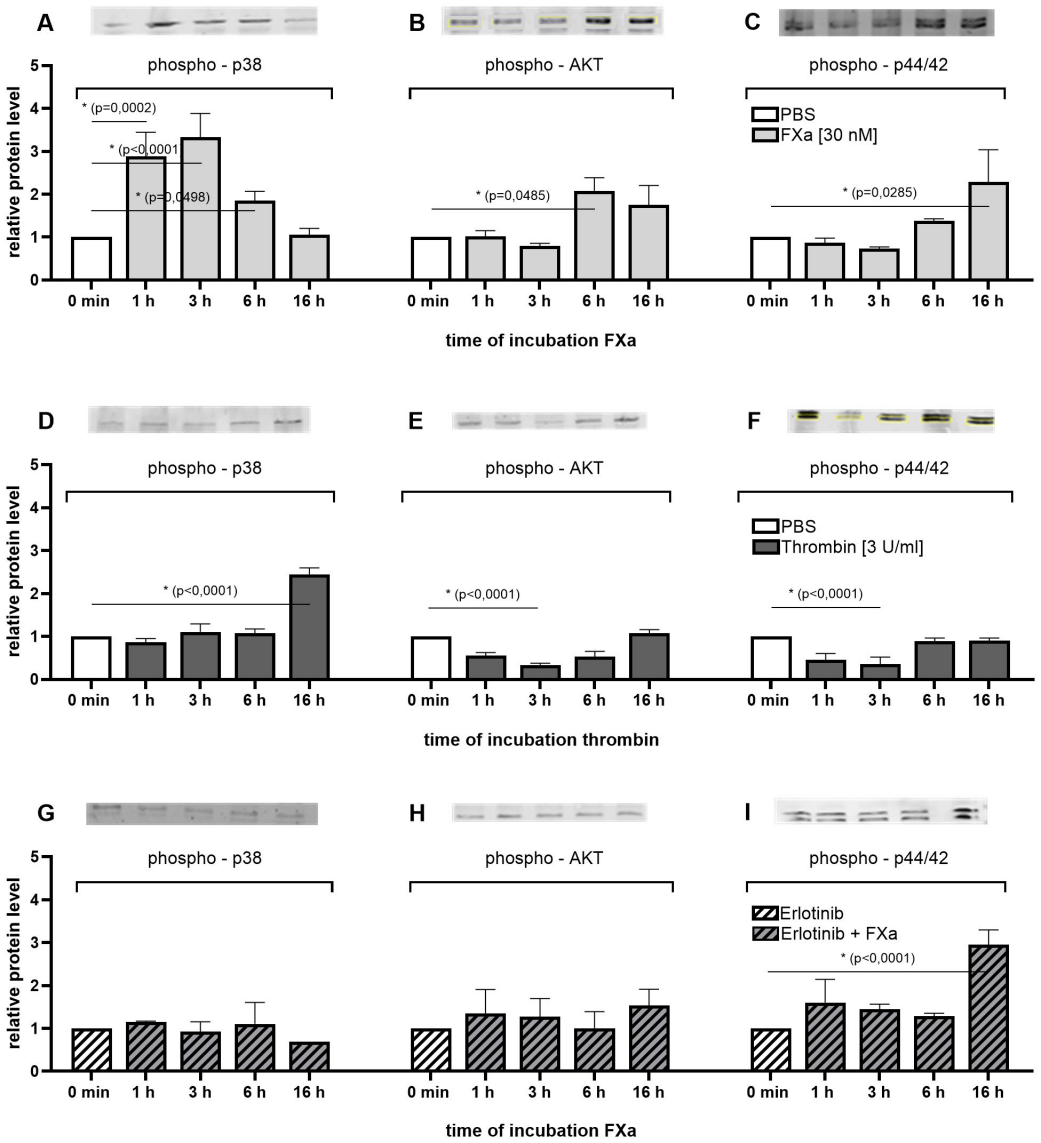
Time-dependent activation of mitogenic pathways in the MC38 cell line after treatment with coagulation factors with or without EGFR inhibition. Western Blot analyses demonstrate the relative changes in the expression of phosphorylated p38, AKT and p44/42 MAPK after treatment with FXa **(A–C)**, thrombin **(D–F)** or EGFR inhibition prior FXa treatment **(G–I)** for up to 16 h. Equal amounts of protein were loaded. Phosphorylated signals were normalized to endogenous levels of total p38, AKT or p44/42 MAPK, as well as the amount of total protein or β-Actin per lane. Shown is the fold induction relative to control. Mean ± SD of n independent experiments is presented for all data. n =3-6, One-way ANOVA, Dunnett posthoc test, p < 0.05 (*).

The protein levels of activated and thus phosphorylated p38, AKT and p44/42 were significantly increased over time following FXa stimulation (**Figures 2A–C**). Short-term incubation notably increased p38 phosphorylation up to 4 -fold (**Figure 2A**), whereas long-term FXa incubation led to a 2-fold increase in p44/42 and AKT signaling (**Figures 2B, C**) compared to the untreated control. Both p44/42 (also known as Erk 1/2) and p38 are mitogen-activated protein kinases (MAPK) whose activation can trigger proliferation and motility (23). AKT (also known as PKB or Rac) is involved in PI3K signaling and plays a critical role in cell survival and apoptosis (24).

Inhibition of EGFR with Erlotinib, a selective inhibitor of the tyrosine kinase domain of the EGF receptor, prior to incubation with FXa fully suppressed the activation of p38 and AKT (**Figures 2G, H**). Following pre-incubation of the cells with Erlotinib, FXa stimulation up to 16 h did not induce an increase in phosphorylation of p38 and AKT. The activation levels of p38 and AKT in the stimulated approaches are equivalent to the basal activation level of the unstimulated control. In the absence of EGFR inhibition by Erlotinib, there is a significant increase in the activation of p38 in response to short-term stimulation by FXa. Conversely, AKT is significantly more activated by long-term stimulation by the activated coagulation factor. Elotinib has been demonstrated to completely inhibit both effects. This suggests an EGFR-dependent co-signaling in p38 MAPK- and AKT-PI3K-signal pathways in these cells. Interestingly, inhibition of EGFR in murine MC38 cells did not impact the relative levels of phosphorylated p44/42 protein (**Figure 2I**). Treatment of the cells with FXa resulted in increased phosphorylation of p44/42 after 16 h, both without (**Figure 2C**) and with (**Figure 2I**) pre-EGFR inhibition.

Compared to the incubation of the colon cancer cells with FXa over time, the incubation with thrombin resulted in a greatly delayed p38 phosphorylation and an attenuated phosphorylation of AKT and p44/42 (**Figures 2D–F**). This observation suggests an increased sensitivity of the cells to FXa compared to thrombin.

**Figure 3:**
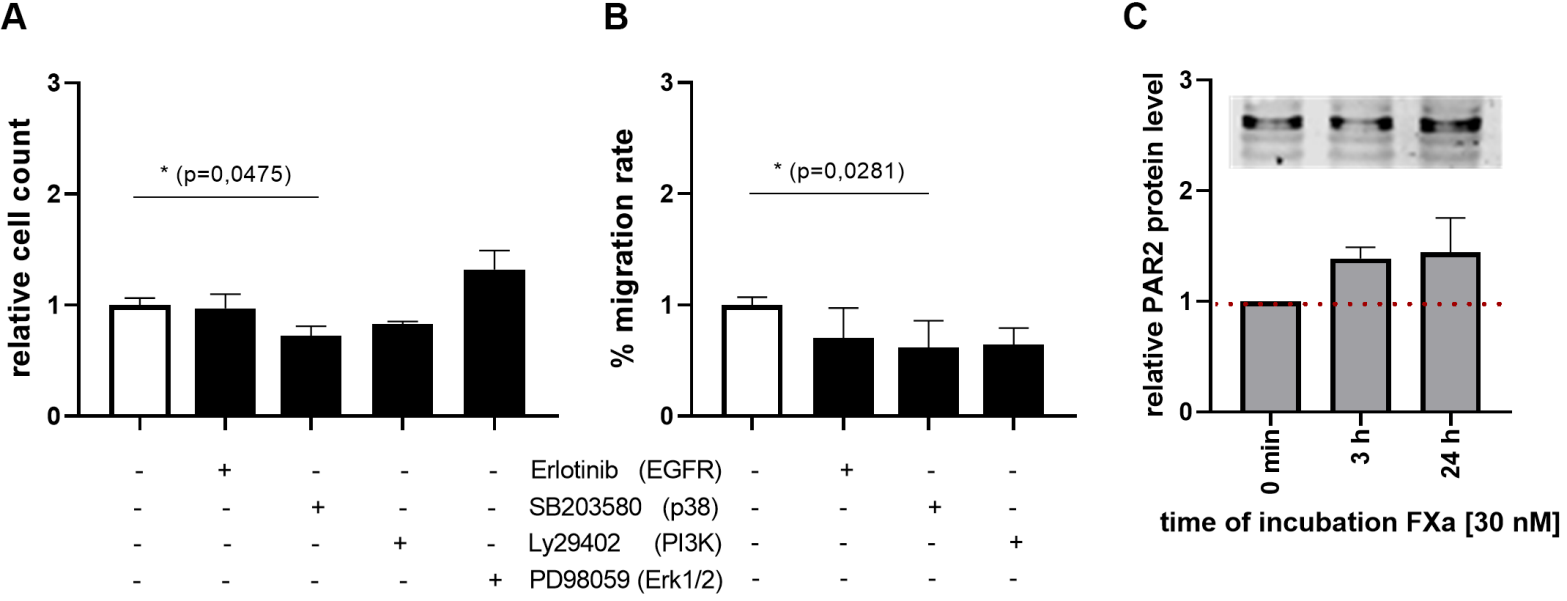
P38 MAPK, PI3K and EGFR signaling in proliferation and migration of MC38 cell line. The effects on proliferation and migration of the murine MC38 cells were evaluated through the use of specific inhibitors. The inhibited protein is indicated in brackets. Inhibition of p38 MAPK significantly decreased proliferation and migration rates of MC38 cells. PI3K and EGFR inhibition also resulted in reduced migration rates. PAR2 expression was analyzed via Western Blotting (M) The expression was normalized to total protein or β-Actin per lane. Shown is the fold induction relative to control. 30 nM FXa induced PAR2 protein level. Mean ± SD of n independent experiments is presented for all data. N =3-6, One-way ANOVA, Dunnett posthoc test, p < 0.05 (*).

Subsequent investigations demonstrated that the inhibition of p38 by SB203580 has a marked inhibitory effect on both proliferation and migration of murine MC38 cells (**Figures 3A, B**). Inhibition of PI3-kinase also resulted in a reduction in the proliferative and migratory abilities of the cells (**Figures 3A, B**). The inhibition of EGFR using Erlotinib, led to a reduction in the cells’ ability to migrate (**Figure 3B**). The responses of MC38 cells to FXa appear to be attributed to PAR2 expression in these cells. Moreover, Western blot analyses demonstrated that PAR2 protein-expression can be modestly elevated by FXa stimulation (**Figure 3C**).

### 3.3 PAR2 deficiency attenuates tumor progression *in vivo*

The second part of the study aimed to analyze the effect of FXa inhibition and PAR2 expression *in vivo* using a subcutaneous mouse tumor model. The murine MC38 cells were injected subcutaneously into (WT mice and PAR2-KO mice on a C57Bl/6 background. The mice, aged 8–10 weeks, were divided into three groups: control (vehicle only) and two Apixaban treatment groups (including vehicle). The direct FXa inhibitor Apixaban was orally administered daily at 5 mg/kg body weight or 50 mg/kg body weight while the control group received a vehicle only solution orally on a daily basis.

**Figure 4:**
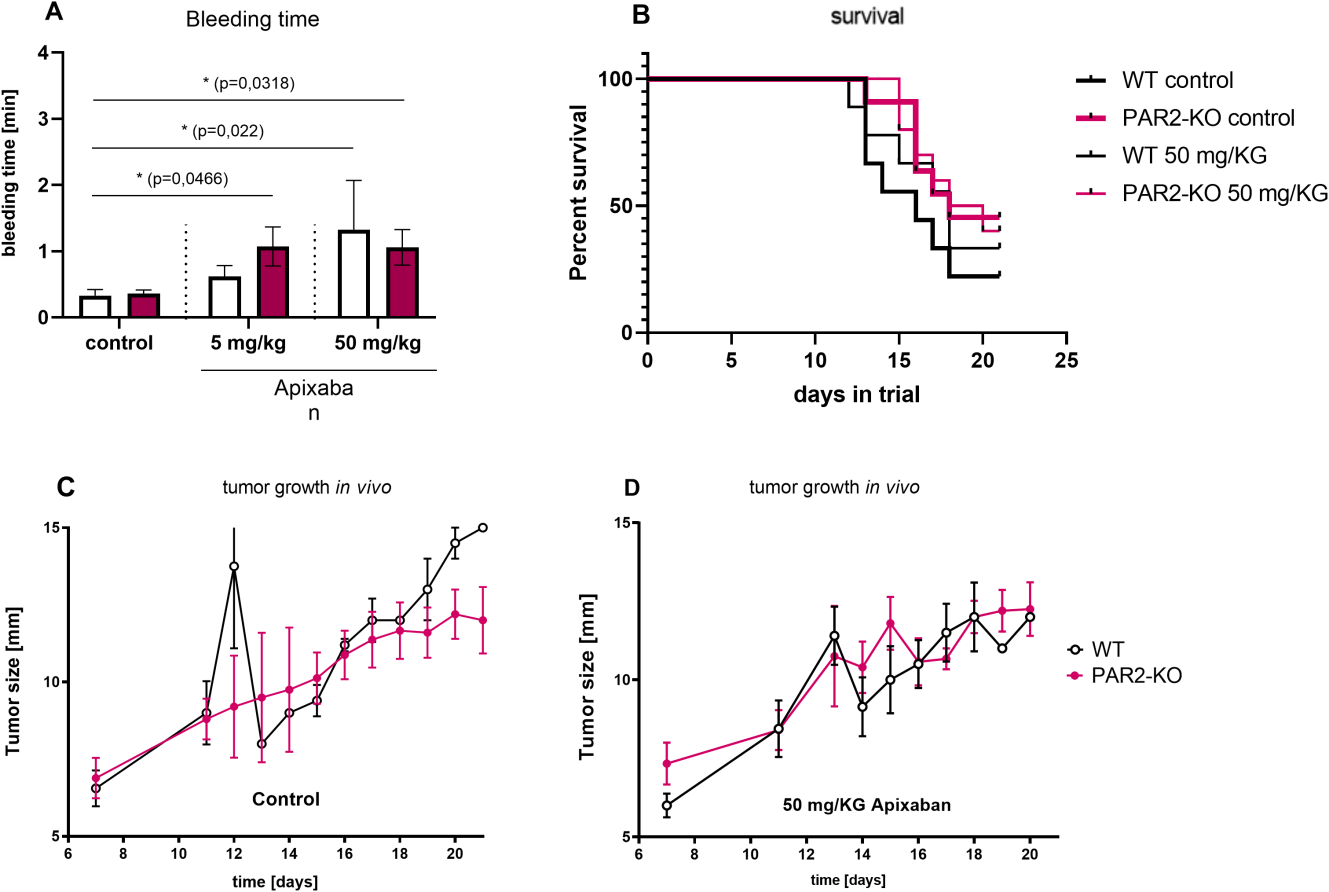
Characteristic *in vivo* values. Tail bleeding assay. **(A)** The direct FXa inhibitor Apixaban prolonged bleeding time significantly. Time was measured in mice that received 5 mg/kg Apixaban, 50 mg/kg Apixaban, or vehicle only (control) in both WT (white) and PAR2-KO (pink) mice. Mean ± SD of n independent experiments is presented for all data. n =5-6, Two-way ANOVA, p < 0.05 (*). Survival. **(B)** Kaplan-Meier estimates for treated (narrow line) and untreated (wide line) WT (black) and PAR2-KO (pink) mice. The survival duration of PAR2-KO animals was found to be significantly longer than that of WT animals. Apixaban did not demonstrate a substantial impact on this survival difference. Tumor growth *in vivo*. Untreated **(C)** and treated **(D)** WT (black) and PAR2-KO (pink) animals measured for maximum tumor extension. Apixaban demonstrates no significant effect.

Tumor growth was observed over a period of 21 days. Apixaban treatment demonstrated no significant impact on tumor progression (**Figures 4C, D**). A tail bleeding assay was performed to ascertain whether the direct FXa inhibitor had entered the systemic circulation and demonstrated the ability to prolong bleeding time. The above test involved clipping off the animal’s tail tip and measuring the duration for cessation of blood flow. Apixaban treatment resulted in a prolongation of bleeding time in WT and PAR2-KO mice (**Figure 4A**). This effect was more pronounced in PAR2-KO animals than WT and was significant compared to the control animals that received vehicle only and not Apixaban.

The duration of the animal trial was 21 days. If the health status of the test animals deteriorated, they were observed more closely prior to being withdrawn from the study. As demonstrated in **Figure 4B**, PAR2-KO mice had significantly fewer withdrawals from the study compared to their WT counterparts. The condition of the PAR2-KO animals in the experiment was significantly better than that of the WT animals. Administration of Apixaban resulted in a modest improvement in survival in WT mice (**Figure 4B**). PAR2-KO animals did not show any additional benefit from the treatment with the direct FXa inhibitor.

**Figure 5:**
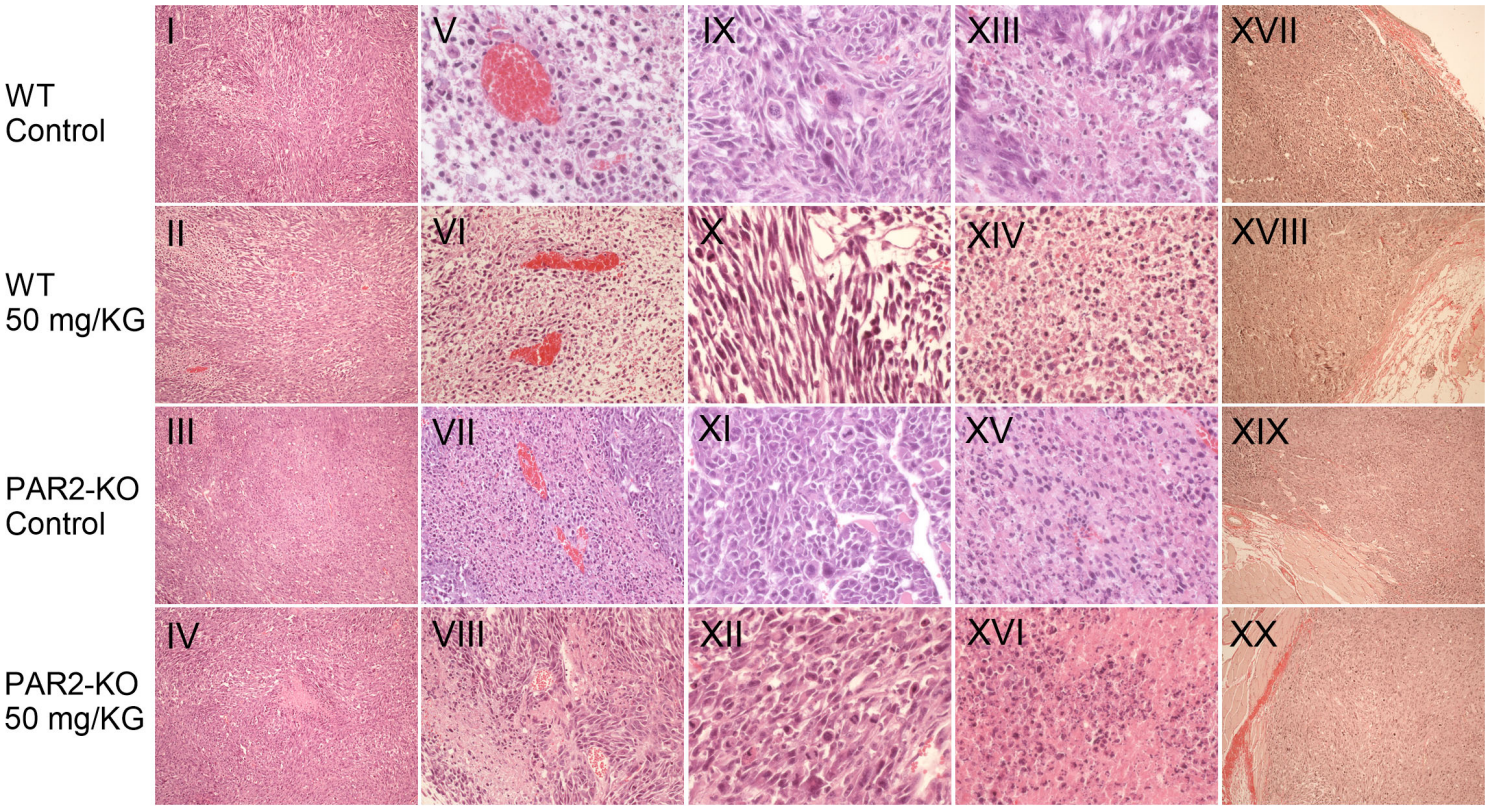
Characteristic *in vivo* values. Staining of resected tumors. Representative images depicting histological features of MC38 tumors of WT (row 1-2) and PAR2-KO mice (row 3-4) are shown. Haematoxylin and eosin (H&E) stained MC38 tumor sections were used to create overviews of the tumors in 1.1 mm depth (I-IV), blood vessels in 535 µm depth (V-VIII), mitotic active sections (IX-XII), and apoptotic areas in 270 µm (XIII-XVI). Hematoxylin stains all basophilic structures, such as the cell nucleus, blue, while eosin stains all basic structures red. These include the cytoplasm and extracellular components. A Pikro-Sirius red staining was performed at a depth of 535 µm to stain collagen fibers at the periphery of the tumors (XVII-XX). Overview staining was photographed at 20x magnification, vessels at 40x magnification, mitotic areas at 200x magnification, apoptotic areas at 100x magnification. The Pikro-Sirius staining as overview staining was magnified 10x.

At the end of the study, organs and tumors were removed and subsequently analyzed. Histological examinations of treated and untreated WT and PAR2-KO mice revealed no discernible differences (**Figure 5**).

**Figure 6:**
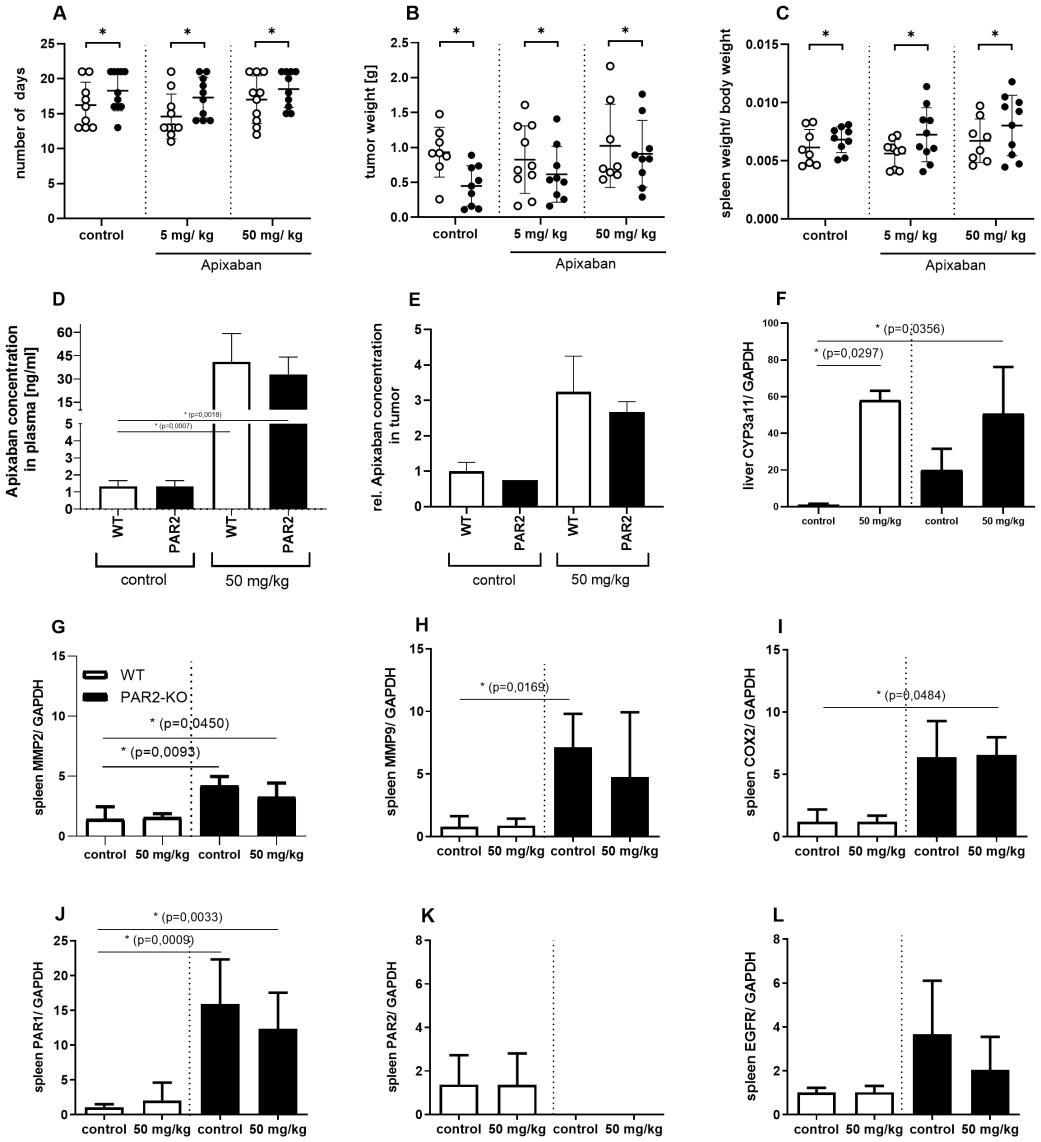
Key figures of the animal study. Trial days. **(A)** Shown is the number of days the animals were subjected to the study, with a maximum of 21 days. PAR2-KO (black) mice stayed significantly longer in the trial than WT (white) mice. This finding is applicable to all three treatment groups. The animals were administered either 5 mg/kg or 50 mg/kg body weight Apixaban plus vehicle or vehicle only (control). Tumor size. **(B)** Additionally, PAR2-KO mice exhibited a significantly reduced tumor size when compared to their WT counterparts. Ratio of spleen weight to body weight. **(C)** Macroscopic discrepancies between the animal strains were observed in the ratio of spleen weight to body weight. Compared to WT (white) animals, PAR2-KO (black) animals showed a significantly higher spleen weight in relation to body weight. Apixaban concentration. **(D)** Apixaban plasma concentrations measured by UPLC-MS/MS in WT (white) and PAR2-KO mice (black) treated with 50 mg/kg or w/o (control) Apixaban. **(E)** Significantly higher Apixaban levels could also be detected in the tumors of treated animals. PCR analyzes. **(F)** Apixaban is metabolized via CYP3A4. The murine counterpart is CYP3A11. PCR analyses show that the treatment of the animals with Apixaban significantly increases the expression of CYP3A11 in the liver at RNA level in both, WT (white) and PAR2-KO mice (black). Further Specific RNA analyses of the removed spleens demonstrate elevated expression levels of the following genes in PAR2-KO mice: MMP2 **(G)**, MMP9 **(H)**, COX2 **(I)**, PAR1 **(J)**, EGFR **(L)**. PAR2 can only be detected in WT mice **(K)**. N = 3-6 (I-VI), Shown is the relative expression of the target normalized to Gapdh and relative to the control approaches. Mean ± SD of n independent experiments is presented for all data. One-way ANOVA, Dunnett posthoc test, p < 0.05 (*).

Despite the absence of statistically significant outcomes associated with the administration of Apixaban, the deficiency of PAR2 evidently had a significant influence on tumor progression. The absence of PAR2 not only enhanced the health status of the mice in the study (**Figure 6A**) but also significantly reduced tumor progression (B). Tumors were considerably smaller in PAR2-KO mice in comparison to WT animals (**Figure 6B**). This phenomenon was evident across all three treatment groups (control, 5 mg/kg Apixaban, 50 mg/kg Apixaban).

It is also noteworthy that there are discernible organic differences between the two animal strains. At the end of the study, the spleens were removed and weighed, and the body weights of the animals were determined. The ratios of these parameters of WT and PAR2-KO animals in the different treatment groups are shown in **Figure 6C**. Interestingly, PAR2-KO mice had a larger spleen than their WT counterparts relative to their body weight (**Supplementary Figure S2**). This might suggest an emphasized participation of the immune system in PAR2-KO mice during combating the pathogenesis of the tumor. In comparison, the direct FXa inhibitor Apixaban exhibits no significant effect in the present study design.

In order to ascertain whether the direct FXa inhibitor could be quantified in the blood plasma, and whether it can be detected directly in the tumor tissue, UPLC-MS/MS analyses were established and performed. The results demonstrate that both, treated WT and treated PAR2-KO animals exhibited significantly higher plasma levels of the inhibitor (only the concentrations for 50 mg/kg Apixaban are shown here) in comparison to the untreated control animals (**Figure 6D**). Concurrently, a significant increase in Apixaban levels was observed within the tumor tissue of treated animals (**Figure 6E**). There are no strain-specific differences between WT and PAR2-KO mice.

In humans, Apixaban is predominantly metabolized by the cytochrome P450 3A4 (CYP3A4) isoenzyme (25). The murine counterpart is CYP3A11. PCR analyses reveal that Apixaban-treated animals show significantly increased expression of CYP3A11 in the liver of both, WT and PAR2-KO mice (**Figure 6F**). Furthermore, PCR analyses reveal the presence of elevated levels of MMP2 (**Figure 6G**), MMP9 (**Figure 6H**) and COX2 (**Figure 6I**), in addition to PAR1 (**Figure 6J**) and EGFR (**Figure 6L**), within the spleens of PAR2-KO mice. The presence of PAR2 was discernible exclusively in the WT group (**Figure 6K**).

## 4 Discussion

Despite the known involvement of the hemostatic system in the processes of cancer growth and metastasis, the underlying molecular mechanisms are not yet fully understood in detail (26).

Coagulation factors may come into contact with the gastrointestinal tract primarily through micro-injuries in the surrounding tissue (27). Furthermore, ectopic expression of FXa in the duodenum has the potential to contribute to the pathophysiology of intestinal adenocarcinomas, as it can spread to other tissues, such as the colon (28). The objective of the present study was thus to examine the correlation between the activated coagulation factor Xa and the progression and severity of colon cancer.

The proliferation-promoting effect of FXa, as described in the literature for fibroblasts and vascular smooth muscle cells *in vitro* (27, 29), could also be demonstrated in the murine colon carcinoma cell line MC38 in the present study. The use of specific FXa inhibitors resulted in a significant reduction in the proliferation rate in the experimental approaches, thereby directly linking the coagulation protease to the observed effect (29). Furthermore, the pro-proliferative effect of FXa in the MC38 cell line could be mimicked and potentiated through selective PAR2 activation. As FXa exerts its cellular effects predominantly via PAR2, this effect could be receptor-dependent. While we were able to observe a significant influence of FXa and the selective activation of PAR2 *in vitro* in our studies, the incubation of the colon carcinoma cells with thrombin or a selective PAR1 agonist was not very efficient. Thrombin did not stimulate marked proliferation or migration in the cells. Compared to FXa, the phosphorylation of p38 was also significantly delayed. This seems to indicate a higher sensitivity of the cells to FXa and fits physiologically with the description of a pronounced expression of PAR2 in the gastrointestinal tract (9).

The activation of protease-activated receptors has been most closely associated with an increase in cell migration in various cell types (30, 31). In our study, selective activation of PAR2, in a comparable manner to stimulation with FXa, exhibits a pronounced pro-migratory influence on the colon carcinoma cells used. While undirected migration entails random movement behavior, directed migration demonstrates targeted movement in response to a chemotactic stimulus (32, 33). Both of these phenomena crucially participate in the pathogenesis of carcinomas. In contrast, thrombin or PAR1 activation had no significant effect. From a pathophysiological perspective, the capacity for migration is a critical determinant in the development of metastases in tumors. The occurrence of this process is associated with a notable deterioration in the prognosis for patients with tumors. The latest studies indicate that coagulation factors exert a pivotal influence on cell migration and associated metastasis (34, 35). The data from our study suggest that FXa and activation of PAR2 may potentially exert a detrimental effect on prognosis in colon carcinoma.

Taken together these findings indicate receptor-dependent effects on proliferation and migration in murine CC cells, as thrombin mediates its cellular effects mainly via PAR1, whereas FXa activates PAR2.

Downstream signaling effects of FXa stimulation may involve transactivation of EGFR with substantial activation of p38 MAPK and PI3K-AKT pathway. These observations are in accordance with the findings from different human colon carcinoma cell lines (7, 36). All three molecular pathways are directly involved in the proliferation and migration of the colon cancer cells used. Activation of the epidermal growth factor receptor (EGFR) plays a pivotal role in invasive processes in colon carcinoma cells and can also influence cell survival (37). Despite the fact that EGFR is overexpressed in numerous carcinomas and thus represents a promising target for therapeutic intervention, mutations of the PI3-kinase or K-Ras result in a lack of response to conventional anti-EGFR therapy (38, 39). It is therefore of utmost importance to identify additional therapeutic target structures that enable future therapies and thus may improve the prognosis of patients.

The data support the involvement of p38 MAPK activation, PI3K-AKT pathway signaling and EGFR transactivation in the proliferation and migration of the murine MC38 cells. The use of specific inhibitors resulted in a significant reduction in the proliferation and migration rate in the experimental approaches after FXa stimulation. The response of the cells to FXa can be attributed to the expression levels of PAR2 in this cell line. Moreover, Western blot analyses demonstrated that incubation with FXa induces an elevated expression of PAR2 protein in the cells. Consequently, FXa can exert a profound influence on cell biological processes via PAR2 signaling. These findings are consistent with those of previous studies that have demonstrated that elevated PAR2 levels are associated with a poorer prognosis for tumor patients (40).

The administration of murine MC38 cells into mice lacking PAR2 resulted in a significant reduction in tumor growth *in vivo* compared to WT animals. In addition, a significantly better health condition was observed in mice with PAR2 deficiency in the sense of a longer endurance under the experimental conditions. Overall, this suggests a pathophysiologically unfavorable role for PAR2 in colon cancer and identifies PAR2 as a potential therapeutic target.

The underlying cause of the enlarged spleens observed in PAR2-KO animals remains unclear at this time. Subsequent analyses did not reveal any differences in the immune cell populations of untreated or treated PAR2-KO or WT animals (data not shown). At this stage this is a limitation of the study. Further research is required in order to analyze immune-mediated antitumor mechanisms in PAR2-KO mice. This effect of an enlarged spleen was particularly evident in female PAR2-KO animals in the present study. Healthy female PAR2-KO mice do not show this effect compared to WT animals. The impact of the murine colon carcinoma cell injection was the only factor that resulted in the manifestation of this effect. In the present study, female PAR2-KO animals exhibited a superior state of health in comparison to their male counterparts and the wild types. Further studies are required to analyze the cause and causal chain of these events.

FXa exerts its cellular effect via PAR2, but the direct FXa inhibitor Apixaban did not significantly attenuate tumor growth *in vivo*. A tail-tip hemorrhage assay was conducted to ascertain the efficacy of Apixaban entering the systemic circulation. As anticipated, the bleeding time in Apixaban-treated WT and PAR2-KO animals was prolonged in comparison to the control animals (without Apixaban). This indicates that the direct FXa inhibitor has reached the systemic circulation in both WT and PAR2-KO animals and exerts an inhibitory influence on blood coagulation. This finding was corroborated by the quantification of Apixaban concentrations in the plasma of the test animals, which revealed that treated animals exhibited significantly higher plasma levels. To ascertain whether Apixaban was incapable of penetrating the tumor and consequently lacked an anti-tumor effect, a methodology for quantifying the Apixaban concentration was established. The results demonstrated that increased Apixaban levels could be measured in the tumors of treated animals compared to the control animals. This shows that although Apixaban reached measurable concentrations within the tumor tissue and in plasma sufficient to influence hemostasis, no significant effect on tumor cell growth was observed. Therefore, the cellular toxicity of the component in the concentrations used here does not appear to be sufficient to achieve cell-damaging effects and inhibit tumor cell growth. It seems likely that the influence of FXa on tumor progression may be less pronounced than that of PAR2 activation. This phenomenon may be attributable to the observation that, in addition to FXa, other mammalian serine proteases have the capacity to proteolytically activate PAR2. These include trypsin, anticoagulant protease activated protein C (aPC), chymase, cathepsin G and S, or neutrophil elastase (41). The measurement of serine protease activity has the potential to serve as an indicator of the activity of these proteases instead of FXa. Furthermore, a variety of cysteine proteases (e.g. calpain-2), metalloproteases (e.g. matrix metalloproteinases, MMPs) and non-mammalian proteases have been shown to induce PAR2 signalling (41). This significant number of PAR2 activators further emphasizes the importance of PAR2 as a promising new target for the treatment of colon cancer. Especially in the context of recent studies, which have identified a common axis of PAR2 and beta-catenin signaling in colon cancer. The Wnt/β-catenin pathway plays an important role in embryonic development. Nevertheless, dysregulation has been demonstrated to be associated with a range of diseases, including cancer (42). Furthermore, the Wnt/β-catenin pathway is a primary driver of therapy resistance (43). PAR2 has been shown to correlate with a poorer prognosis in advanced cancer cases, in addition to contributing to metastasis through the maintenance and expansion of cancer stem-like cells (CSCs). Studies show that PAR2 exerts a regulatory function over CSC self-renewal via the ERK/GSK/beta-catenin signaling pathway (44). The activation of PAR2 has been shown to stabilize beta-catenin, thus increasing the transcription of oncogenic beta-catenin target genes (e.g. c-Myc, cyclin D1), which can promote cell proliferation and survival (43). The investigation of the common signaling pathway of PAR2 and β-catenin is a subject worthy of further attention in subsequent research studies.

### 4.1 Limitation of the study

The *in vivo* model employed is a subcutaneous syngeneic tumor model. This model’s ease of handling, reproducibility, and the simplicity of monitoring tumor growth are advantageous. Furthermore, the absence of major surgical interventions is a major advantage, given the recognized potential of trauma and inflammation to induce non-physiological tumor microenvironments (45, 46). Conversely, this approach is unable to fully reflect the complexity of carcinogenesis. Human tumors evolve over a period of months and years; however, the present study considers a 21-day period. Consequently, potential resistance mechanisms and altered immune microenvironments cannot be mapped identically. Furthermore, subcutaneous tissue differs from gastrointestinal tissue. Notwithstanding these limitations, it is noteworthy that the study successfully demonstrates the capacity to induce tumor growth with a high degree of precision and reproducibility. Moreover, the analysis of therapeutic interventions and, above all, of gene knockouts is a significant strength of the study.

## Data Availability

The raw data supporting the conclusions of this article will be made available by the authors, without undue reservation.
