# Supplementary material for: Significance of FXa and its receptor PAR2 for the growth of colon cancer cells in vitro and in vivo
Source: Front Oncol. 2025 Jul 21;15:1631350. doi: 10.3389/fonc.2025.1631350 (PMC12318734; doi:10.3389/fonc.2025.1631350)
Supplement: Supplementary file 1 [file DataSheet1.pdf]

Supplementary Data

Significance of FXa and its receptor PAR2 for the growth of colon cancer cells *in vitro* and *in vivo*

Ulrike Meyer[1,2]*, Vincent Rönnpagel[2], Sophie Grammbauer[3], Mirjam von Lucadou[4], Ursula Rauch-Kröhnert[5], Edzard Schwedhelm[4], Frank Dombrowski[3], Christoph Ritter[6], Bernhard H. Rauch[1,2]*

[1]Pharmacology and Toxicology, University Medicine Oldenburg, Carl von Ossietzky Universität Oldenburg, Oldenburg, Germany
[2]Department of General Pharmacology, Institute of Pharmacology, University Medicine Greifswald, Greifswald, Germany
[3]Institute of Pathology, University Medicine Greifswald, Greifswald, Germany
[4]University Medical Center Eppendorf, Institute of Clinical Pharmacology and Toxicology, Hamburg, Germany
[5]Department of Cardiology, Angiology and Intensive Care, German Heart Center of Charité, Berlin, Germany
[6]Department of Clinical Pharmacy, Institute of Pharmacy, University Greifswald, Greifswald, Germany

This supplementary file includes:

1. Materials and Methods (additional detailed description)
2. Supplementary figures and corresponding legends
3. Human Data

Material and Methods (additional detailed description)

Animals

The PAR2-KO strain utilized in the present study is commercially available and was originally generated by Schmidlin et al. in 2002.[20] The animals are backcrossed every 10 generations in order to minimize genetic drift. The genetic homozygous knockout of the PAR2 gene (F2rl1) has been confirmed by genotyping (Figure S1). For this purpose, the biopsies from the mice were digested overnight at 56°C prior to the isolation of the DNA. The digested tissue suspensions were then mixed with 300 µl of saturated NaCl solution, after which the samples were subjected to centrifugation at 13,000 rpm at 4°C for 10 minutes. The clear interphase was transferred to a new Eppendorf tube and mixed with 500 µl of isopropanol. Subsequent centrifugation at 13,000 rpm for five minutes produced a pellet that was thoroughly washed with ethanol. Following the drying process, the pellet was resuspended in TE buffer. Subsequently, a polymerase chain reaction (PCR) was performed in order to amplify the DNA from the samples. The required reaction mix comprised DNA, Dream Taq polymerase, Dream Taq buffer, deoxynucleoside triphosphates, water, $MgCl_2$ and the specific forward and reverse primers for WT and PAR2-KO, respectively. The following table presents the sequence of the primers:

Table 1. Sequence of the primers used for PAR2 genotyping

**Sequence 5' → 3'**	**Primer**
TCA AAG ACT GCT GGT GGT TG	WT forward
GGT CCA ACA GTA AGG CTG CT	WT reverse
GCA GCC AAT ATG GGA TCG	PAR2 forward
ATC AGA GCA GCC GAT TGT CT	PAR2 reverse

Following the amplification of the DNA, the samples were subjected to electrophoresis in a 2% agarose gel. The presence of WT bands is anticipated at 345 base pairs (bp), homozygous KO bands at 100 bp, and heterozygous bands at 345 bp and 100 bp. It is imperative to note that the experimental subjects comprised exclusively of homozygous KO animals.

In vivo Tumor Model

Randomization: The offspring of WT and PAR2-KO breeding pairs were divided equally into pairs of two and separated by sex into the two Apixaban treatment groups and the control group. The first two same-sex offspring of a breeding pair were assigned to group 1, the next two to group 2, and so on, in order to minimize familial effects. The allocation of subjects was conducted in pairs, with the objective of maintaining cohousing of siblings of the same sex within the cages. This was done in accordance with ethical principles to ensure the well-being and social needs of the subjects.

Blinding: The Blinding of the study was ensured by the staffing. The technical assistance of the working group was responsible for the selection of the cage sequence in the housing cabinet and the daily release of the animals to the scientist for the examinations. The cages did not contain any information on the group allocation of the animals. Since both WT and PAR2-KO have a C57BL/6J background, the animals cannot be distinguished phenotypically. The scientist was therefore unable to draw any conclusions about the treatment of the individual animals.

Supplementary Figures

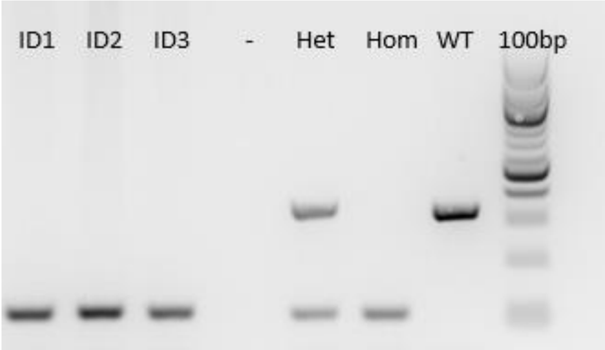

Figure S1. PAR2 Genotyping. The ear punches of three exemplary mice (ID1, ID2 and ID3) were digested and the resulting DNA was amplified prior to being separated on a 2% agarose gel. The expected PCR products are 100 + 345 bp for heterozygous, 100 bp for homozygous KO and 345 bp for WT. WT, heterozygous and homozygous PAR2-KO ear punches were used as a control. A 100 bp marker was used as a standard. All three mice are PAR2-KO. Het: heterozygous; Hom: homozygous KO; WT: wildtype.

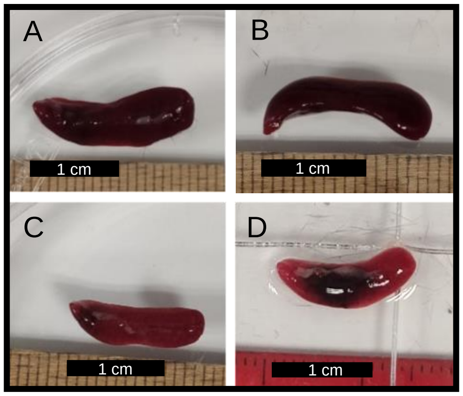

Figure S2. Resected spleens from PAR2-KO and WT mice. The following presentation provides a comprehensive illustration of the removed spleens from both PAR2-KO (A, B) and WT (C, D) mice. The spleens removed were measured and weighed. Subsequent, the spleen weight was normalized to the body weight of the animals. Regardless of the Apixaban treatment, PAR2-KO mice exhibited a higher spleen-to-body weight ratio than the WT animals.

Human Data

Material and Methods

Cell Culture

A comparison of the murine data was conducted on human cells using the commercially available human colon carcinoma cell line HCT116. These cells are derived from the colon of a male Caucasian. The HCT116 cell line was maintained in McCoy's 5a Medium w: 3,0 g/L Glucose, w: stabiles Glutamin, w: 2,0 mM Natriumpyruvat, w: 2,2 g/L NaHCO3 [Cytion, Cat. #820200a] with 10 % FCS [PAN Biotech, Cat. #PANBP30-3306] and utilized until the 27th passage. The cell line was monthly tested for mycoplasma contamination via PCR analysis [Abcam, Cat. #ab289834]. The Cells were stimulated with FXa (0,1 — 30 nM) [Enzo Lifescience, Cat. #BML-SE362-0100] or selective PAR2-activating peptide AP2 (sequence: SLIGRL, 10 — 100 µl) [Bachem AG, Cat. #H-5078] in a time- dependent manner after maintaining the cells in serum-free media for 16 h. Measurements of cell proliferation and migration were conducted according to the established protocols, dedicated in the “Materials and Methods” part of the manuscript.

Results

As demonstrated in Figure S3, the generation time of the human colon carcinoma cell line HCT116 (26.28 h) is longer than the doubling time of the murine MC38 cells (A). After incubation with coagulation factor FXa at concentrations of 30 nM in a time-dependent manner (24 h — 48 h), the growth rate of the human cells was unaffected when compared to the negative control without FXa (Figure S3 B,C). However, the cells react to the FXa stimulus with a significantly increased migration rate (Figure S3 D). This effect can be mimicked through the selective activation of PAR2 by AP2 (E). The effect appears to be mediated in a receptor-dependent manner. These results correspond to the in vitro observations made in the murine MC38 cells. In contrast to the murine cells, no significant activation of p38, AKT or p44/42 can be detected on protein level (F).

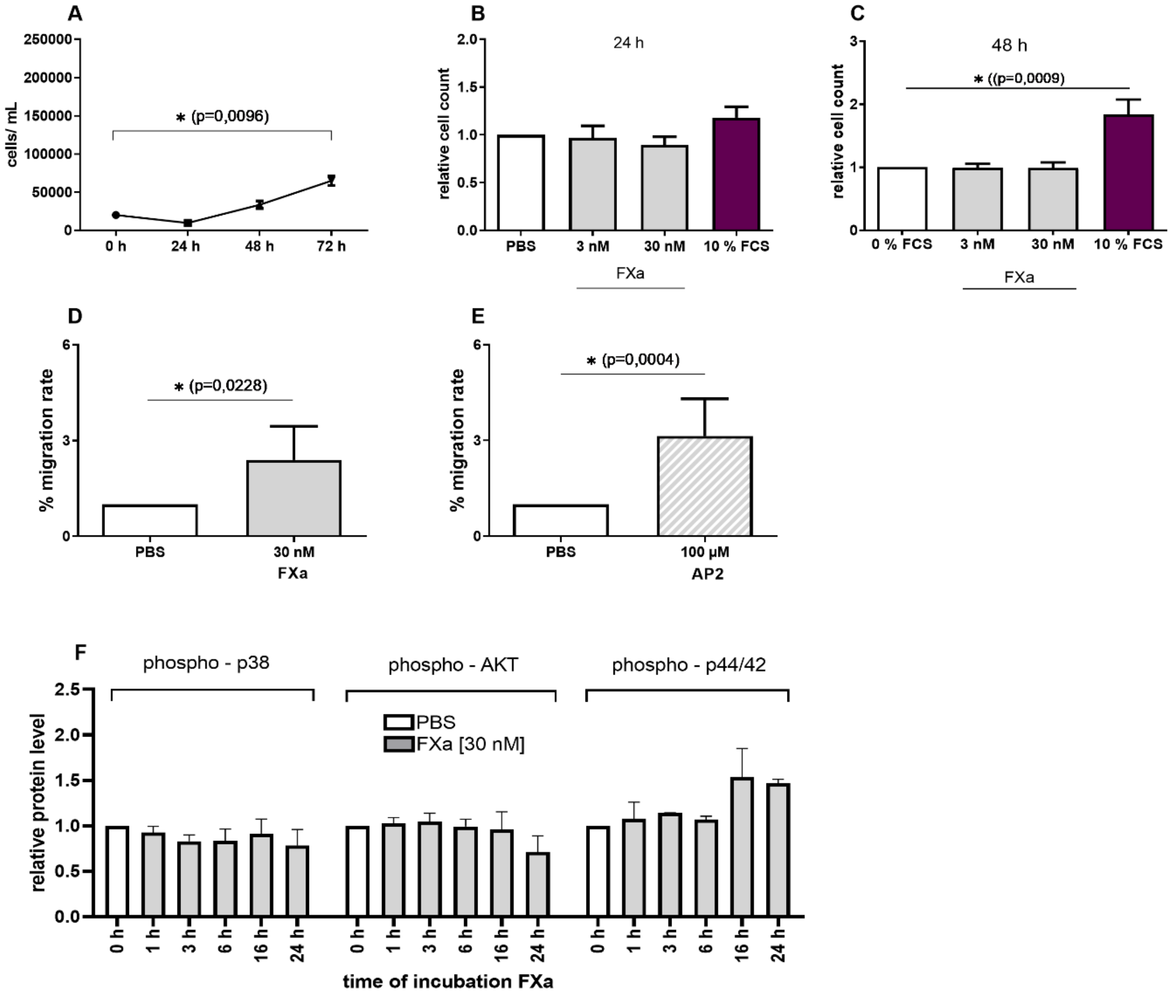

Figure S3. Human in vitro data. In vitro effects of FXa and PAR2 activation on proliferation and migration of human CC cell line HCT116. (A) The basal generation time of the human cell line is 26.28 h. A Stimulation with 30 nM FXa for 24 h (B) or 48 h (C) has no effect on the doubling time. 10 % FCS is the only stimulus significantly inducing proliferation. Mean ± SD of n independent experiments is presented for all data. n =3, One-way ANOVA, Dunnett posthoc test, $p < 0.05$ (*). Migration. In contrast, FXa (D) and selective PAR2 activation using AP2 (E) significantly increased cell migration. n =6-7, t-test, $p < 0.05$ (*). The time-dependent activation of typical mitogenic pathways was analyzed on protein level. Western Blot analyses demonstrate no significant relative changes in the expression of phosphorylated p38, AKT and p44/42 MAPK after treatment with FXa for up to 24 h. Equal amounts of protein were loaded. Phosphorylated signals were normalized to endogenous levels of total p38, AKT or p44/42 MAPK, as well as the amount of total protein or β-Actin per lane. Shown is the fold induction relative to control. Mean ± SD of n independent experiments is presented for all data. n =6, One-way ANOVA, Dunnett posthoc test, $p < 0.05$ (*).

Western Blot

Figure 2A. MC38 FXa p38

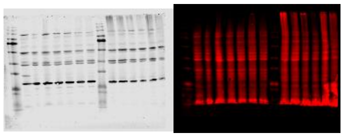

Figure 2B. MC38 FXa AKT

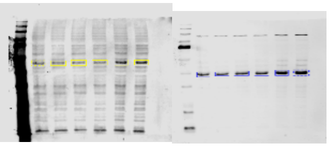

Figure 2C MC38 FXa p44/42

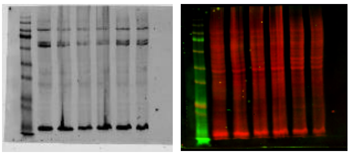

Figure 2D. MC38 Thrombin p38

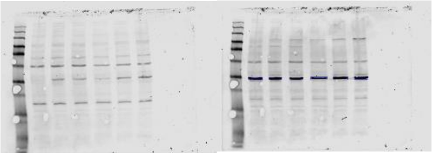

Figure 2E.MC38 Thrombin AKT

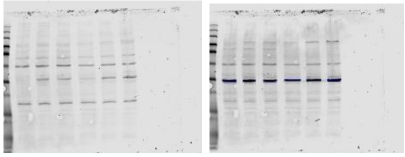

Figure 2F. MC38 Thrombin p44/42

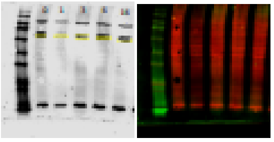

Figure 2G. MC38 Erlotinib+FXa p38

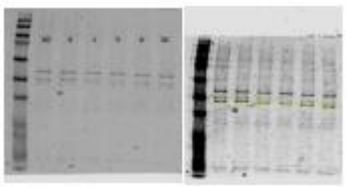

Figure 2H.MC38 Erlotinib+FXa AKT

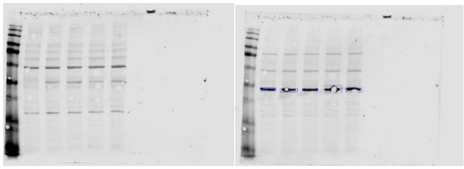

Figure 2I. MC38 Erlotinib+FXa p44/42

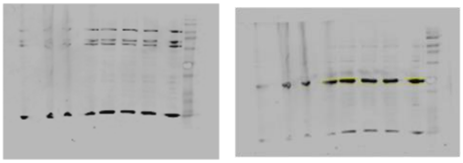

Figure 2M.MC38 FXa PAR2

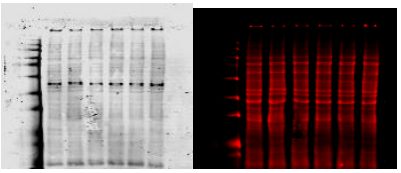

Figure S4.Time-dependent activation of mitogenic pathways in the MC38 cell line after treatment with coagulation factors FXa or Thrombin with or without EGFR inhibition. Shown are the raw Western Blot images. Protein marker VI was used as a standard. Total protein staining or β-Actin was used as reference staining.
